# A Novel Artificial Immune Algorithm for Spatial Clustering with Obstacle Constraint and Its Applications

**DOI:** 10.1155/2014/160730

**Published:** 2014-11-04

**Authors:** Liping Sun, Yonglong Luo, Xintao Ding, Ji Zhang

**Affiliations:** ^1^College of National Territorial Resources and Tourism, Anhui Normal University, China; ^2^Engineering Technology Research Center of Network and Information Security, Anhui Normal University, China; ^3^Faculty of Health, Engineering and Sciences, University of Southern Queensland, Australia

## Abstract

An important component of a spatial clustering algorithm is the distance measure between sample points in object space. In this paper, the traditional Euclidean distance measure is replaced with innovative obstacle distance measure for spatial clustering under obstacle constraints. Firstly, we present a path searching algorithm to approximate the obstacle distance between two points for dealing with obstacles and facilitators. Taking obstacle distance as similarity metric, we subsequently propose the artificial immune clustering with obstacle entity (AICOE) algorithm for clustering spatial point data in the presence of obstacles and facilitators. Finally, the paper presents a comparative analysis of AICOE algorithm and the classical clustering algorithms. Our clustering model based on artificial immune system is also applied to the case of public facility location problem in order to establish the practical applicability of our approach. By using the clone selection principle and updating the cluster centers based on the elite antibodies, the AICOE algorithm is able to achieve the global optimum and better clustering effect.

## 1. Introduction

Spatial clustering analysis is an important research problem in data mining and knowledge discovery, the aim of which is to group spatial data points into clusters. Based on the similarity or spatial proximity of spatial entities, the spatial dataset is divided into a series of meaningful clusters [1]. Due to the spatial data cluster rule, clustering algorithms can be divided into spatial clustering algorithm based on partition [2, 3], spatial clustering algorithm based on hierarchy [4, 5], spatial clustering algorithm based on density [6], and spatial clustering algorithm based on grid [7].

**Figure 1:**
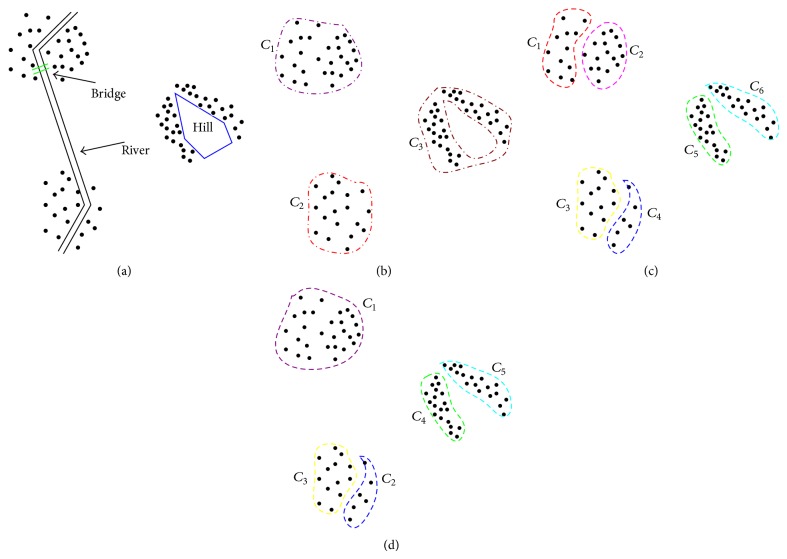
Spatial clustering with obstacle and facilitator constraints: (a) spatial dataset with obstacles; (b) spatial clustering result ignoring obstacles; (c) spatial clustering result considering obstacles; (d) spatial clustering result considering both obstacles and facilitators.

The distance measure between sample points in object space is an important component of a spatial clustering algorithm. The above traditional clustering algorithms assume that two spatial entities are directly reachable and use a variety of straight-line distance metrics to measure the degree of similarity between spatial entities. However physical barriers often exist in the realistic region. If these obstacles and facilitators are not considered during the clustering process, the clustering results are often not realistic. Taking the simulated dataset in Figure 1(a) as an example, where the points represent the location of consumers, the clustering result shown in Figure 1(b) can be obtained, when the rivers and hill as obstacles are not considered. If the obstacles are taken into account and bridges as facilitators are not considered, the clustering result in Figure 1(c) can be gained. Considering both the obstacles and facilitators, Figure 1(d) demonstrates the more efficient clustering patterns.

At present, only a few clustering algorithms consider obstacles and/or facilitators in the spatial clustering process. COE-CLARANS algorithm [8] is the first spatial clustering algorithm with obstacles constraints in a spatial database, which is an extension of classic partitional clustering algorithm. It has similar limitations to the CLARANS algorithm [9], which has sensitive density variation and poor efficiency. DBCluC [10] extends the concepts of DBSCAN algorithm [11], utilizing obstruction lines to fill the visible space of obstacles. However, it cannot discover clusters of different densities. DBRS+ is the extension of DBRS algorithm [12], considering the continuity in a neighborhood. Global parameters used by DBRS+ algorithm make it suffer from the problem of uneven density. AUTOCLUST+ is a graph-based clustering algorithm, which is based on AUTOCLUST clustering algorithm [13]. For the statistical indicators used by AUTOCLUST+ algorithm, it could not deal with planar obstacles. Liu et al. presented an adaptive spatial clustering algorithm [14] in the presence of obstacles and facilitators, which has the same defect as AUTOCLUST+ algorithm.

Recently, the artificial immune system (AIS) inspired by biological evolution provides a new idea for clustering analysis. Due to the adaptability and self-organising behaviour of the artificial immune system, it has gradually become a research hotspot in the domain of smart computing [15–20]. Bereta and Burczyński performed the clustering analysis by means of an effective and stable immune *K*-means algorithm for both unsupervised and supervised learning [21]. Gou et al. proposed the multielitist immune clonal quantum clustering algorithm by embedding a potential evolution formula into affinity function calculation of multielitist immune clonal optimization and updating the cluster center based on the distance matrix [22]. Liu et al. put forward a novel immune clustering algorithm based on clonal selection method and immunodominance theory [23].

In this paper, a path searching algorithm is firstly proposed for the approximate optimal path between two points among obstacles to achieve the corresponding obstacle distance. It does not need preprocessing and can deal with both linear and planar obstacles. Based on the path searching algorithm, a spatial clustering algorithm is proposed to the spatial data clustering in the presence of both obstacles and facilitators. A case study is also carried out to apply our method to the problem of public facility optimization.

The remainder of this paper is organized as follows.  Section 2 at first presents the path searching algorithm and then elaborates the details of AICOE algorithm, including analysis of population partition, the design of affinity function, and immune operators.  Section 3 shows the experimental results.  Section 4 presents the conclusions and main findings.

## 2. Theoretical Framework

### 2.1. Obstacles Representation

Physical obstacles in the real world can generally be divided into linear obstacles (e.g., river, highway) and planar obstacles (e.g., lake). Facilitators (e.g., bridge) are physical objects which can strengthen straight reachability among objects. In processing geospatial data, representation of the spatial entities needs to be firstly determined [14]. In this paper, the vector data structure is used to represent spatial data. Obstacles entities are approximated as polylines and polygons. A facilitator is abstracted as a vertex on an obstacle.

Relevant definitions are provided as follows.

**Definition 1 (linear obstacles).**

Let *L* = {*L*_*i*_∣*L*_*i*_ = (*V*_*i*_^(*L*)^, *E*_*i*_^(*L*)^), *i* ∈ *Z* ^+^} be polyline obstacles set, where *V*_*i*_^(*L*)^ is the set of vertices of *L*_*i*_; *E*_*i*_^(*L*)^ = {(*v*_*i*_*k*__, *v*_*i*_*k*+1__)∣*v*_*i*_*k*__, *v*_*i*_*k*+1__ ∈ *V*_*i*_^(*L*)^, *v*_*i*_*k*__ is the adjacent vertex of *v*_*i*_*k*+1__, *k* = 1,…, *M*_*i*_ − 1, *M*_*i*_ is the number of *V*_*i*_^(*L*)^}.

**Definition 2 (planar obstacles).**

Let *S* = {*S*_*i*_∣*S*_*i*_ = (*V*_*i*_^(*S*)^, *E*_*i*_^(*S*)^), *i* ∈ *Z* ^+^} be polygon obstacles set, where *V*_*i*_^(*S*)^ is the set of vertices of *S*_*i*_; *E*_*i*_^(*S*)^ = {(*v*_*i*_*k*__, *v*_*i*_(*k*+1)mod⁡*N*_*i*___)∣*v*_*i*_*k*__, *v*_*i*_(*k*+1)mod⁡*N*_*i*___ ∈ *V*_*i*_^(*S*)^, *v*_*i*_*k*__ is the adjacent vertex of *v*_*i*_(*k*+1)mod⁡|*N*_*i*_|__, *k* = 1,…, *N*_*i*_, *N*_*i*_ is the number of *V*_*i*_^(*S*)^}.

**Definition 3 (facilitators).**

Let *V*_*c*_ = {*V*_*i*_^(*C*)^∣*V*_*i*_^(*C*)^ is the set of facilitators on the *i*th obstacle}.

**Definition 4 (direct reachability).**

For any two points *p*, *q* in a two-dimensional space, *p* is called directly reachable from *q*, if segment *pq* does not intersect with any obstacle; otherwise, *p* is called indirectly reachable from *q*.

### 2.2. The Obstacle Distance between the Spatial Entities

**Figure 2:**
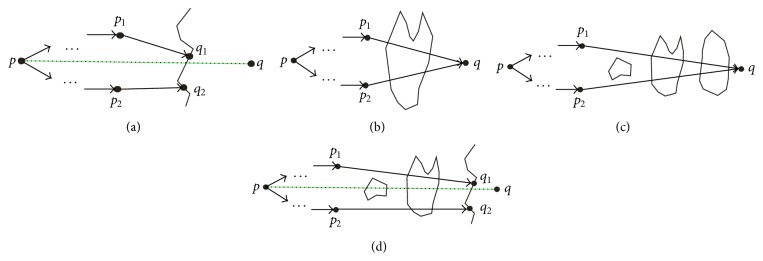
Construction of approximate optimal path between two points with obstacle constraints: (a) intersect with a linear obstacle; (b) intersect with the last planar obstacle; (c) intersect with a planar obstacle and obstacles behind it are all planar; (d) intersect with a planar obstacle and the obstacle behind it is linear.

Currently, the method of distance calculation often computes Euclidean distance between two clustering points. When physical obstacles exist in the real space, obstacles constraints should be taken into account to solve the distance between the two entities in the space. The algorithm handles linear obstacles and planar obstacles, respectively. When traversing linear obstacles, facilitators are also taken into account for path construction.  Figure 2(a) illustrates the process of constructing approximate optimal path for linear obstacle, which presents a schematic view of Step 4 of the algorithm. When traversing planar obstacles, path is generated by the method to construct the minimum convex hull. In the case of no more than 100,000 two-dimensional space data samples, the calculation of the minimum convex hull can be finished within a few seconds [24]. Here Graham algorithm is used to produce the minimum convex hull [25]. Figures 2(b) and 2(c) and Figure 2(d), respectively, illustrate the construction process of the approximate optimal path for planar obstacles.  Figure 2(b) shows a schematic view of the first case of Step 5. Figures 2(c) and 2(d) demonstrate a schematic view of the second case of Step 5.

For the sake of easy presentation of the path searching algorithm, the relevant symbols are defined as follows. Let *o*_*i*_ ∈ *L* ∪ *S* be an obstacle, and $V_{i}^{\left(l\right)}(\vec{pq})\subset V_{c}$ is the vertex subset of *o*_*i*_ on your left hand when you walk along vector $\vec{pq}$ from point *p* to *q*. Similarly, $V_{i}^{\left(r\right)}(\vec{pq})\subset V_{c}$ is the vertex subset of *o*_*i*_ on the right hand. *Gra*(*U*, *p*, *q*) is the smallest convex hull which is constructed from the start point *p* to the end point *q* containing all the points of the vertex set *U*. *Path* ^(*c*)^(*Gra*(*U*, *p*, *q*)) denotes the path from the start point *p* to the end point *q*, which is constructed by the adjacent edges of *Gra*(*U*, *p*, *q*) in the clockwise direction; *Path* ^(*cc*)^(*Gra*(*U*, *p*, *q*)) denotes the path from the start point *p* to the end point *q*, which is constructed by the adjacent edges of *Gra*(*U*, *p*, *q*) in the counterclockwise direction. *path*1 and *path*2, respectively, are the obstacle paths on the left and right hand of $\vec{pq}$. When new segments are added to *path*1 and *path*2, the start points of the added segments are denoted by *p*1 and *p*2, respectively. Similarly, the end points are denoted by *q*1 and *q*2. *d*_*o*_(*p*, *q*) represents the obstacle distance between two spatial entities. If *p* is directly reachablefrom *q*, *d*_*o*_(*p*, *q*) is Euclidean distance between the two points, denoted by *d*(*p*, *q*); if *p* is indirectly reachablefrom *q*, path is configured to bypass the obstacles while *p*, *q*, respectively, are taken as the start and end points.

The path searching algorithm for the approximate optimal path between two points among obstacles can be elaborated as follows.

*Step  1*. If *p* is directly reachable from *q*, then *d*_*o*_(*p*, *q*) = *d*(*p*, *q*), and the algorithm is terminated; otherwise, go to Step 2.

*Step  2*. Find the obstacles intersect with $\vec{pq}$, which in turn are represented as *o*_1_, *o*_2_,…, *o*_*m*_ ∈ *L* ∪ *S*, where *m* is the number of the obstacles.

*Step  3*. Consider *path*1 = *ϕ*, *path*2 = *ϕ*, *p*1 = *p*2 = *p*, and *i* = 0.

*Step  4*. If *o*_*i*_ ∈ *L*, execute the following steps.

- Select the vertex $u\in V_{i}^{\left(l\right)}(\vec{pq})$ which has the smallest distance to $\vec{pq}$.
- Select the vertex $v\in V_{i}^{\left(r\right)}(\vec{pq})$ which has the smallest distance to $\vec{pq}$.
- Consider *q*_1_ = *u*, *q*_2_ = *v*, $path1=path1\cup \vec{p_{1}q_{1}}$, and $path2=path2\cup \vec{p_{2}q_{2}}$.
- Consider *i* + +, *p*_1_ = *q*_1_, and *p*_2_ = *q*_2_.
- Go to Step 6.

*Step  5*. If *o*_*i*_ ∈ *S*, there are the following two cases.

- If *i* = = *m*, execute the following steps.
  - If $\vec{p_{1}q}$ intersects with *o*_*i*_, add $V_{i}^{\left(l\right)}(\vec{p_{1}q})$ to *U*_1_, *path*1 = *path*1 ∪ *Path* ^(*c*)^(*Gra*(*U*_1_, *p*_1_, *q*)).
  - If $\vec{p_{2}q}$ intersects with *o*_*i*_, add $V_{i}^{\left(r\right)}(\vec{p_{2}q})$ to *U*_2_, *path*2 = *path*2∪*Path* ^(*cc*)^(*Gra*(*U*_2_, *p*_2_, *q*)).
  - Consider *i* + +, *p*_1_ = *q*, and *p*_2_ = *q*.
  - Go to Step  6.
- If *i* < *m*, execute the following steps.
  - If *o*_*k*_(*k* = *i*, *i* + 1,…, *m*) ∈ *S*, execute the following steps.
    - Add $V_{k}^{\left(l\right)}(\vec{p_{1}q})$ to *U*_1_, *path*1 = *path*1 ∪ *Path* ^(*c*)^(*Gra*(*U*_1_, *p*_1_, *q*)).
    - Add $V_{k}^{\left(r\right)}(\vec{p_{2}q})$ to *U*_2_, *path*2 = *path*2 ∪ *Path* ^(*cc*)^(*Gra*(*U*_2_, *p*_2_, *q*)).
    - Consider *i* = *m*, *p*_1_ = *q*, and *p*_2_ = *q*.
  - If *o*_*i*_, *o*_*i*+1_,…, *o*_*k*_(*k* < *m*) ∈ *S* and *o*_*k*+1_ ∈ *L*, execute the following steps.
    - Select the vertex $u\in V_{k+1}^{\left(l\right)}(\vec{pq})$ which has the smallest distance to $\vec{pq}$.
    - Select the vertex $v\in V_{k+1}^{\left(r\right)}(\vec{pq})$ which has the smallest distance to $\vec{pq}$.
    - Consider *q*_1_ = *u* and *q*_2_ = *v*.
    - Add $V_{i}^{\left(l\right)}\left(\vec{p_{1}q_{1}}\right),V_{i+1}^{\left(l\right)}\left(\vec{p_{1}q_{1}}\right),\ldots ,V_{k}^{\left(l\right)}(\vec{p_{1}q_{1}})$ to *U*_1_. Consider *path*1 = *path*1∪*Path* ^(*c*)^(*Gra*(*U*_1_, *p*_1_, *q*_1_)).
    - Add $V_{i}^{\left(r\right)}(\vec{p_{2}q_{2}}),V_{i+1}^{\left(r\right)}(\vec{p_{2}q_{2}}),\ldots ,V_{k}^{\left(r\right)}(\vec{p_{2}q_{2}})$ to *U*_2_. Consider *path*2 = *path*2∪*Path* ^(*cc*)^(*Gra*(*U*_2_, *p*_2_, *q*_2_)).
    - Consider *i* = *k* + 1, *p*_1_ = *q*_1_, and *p*_2_ = *q*_2_.

*Step  6*. If *i* < *m*, go to Step  4; otherwise if *p*_1_! = *q* and *p*_2_! = *q*, then $path1=path1\cup \vec{p_{1}q}$, $path2=path2\cup \vec{p_{2}q}\cdot d_{o}(p,q)=min(length(path1),length(path2))$.

### 2.3. Spatial Clustering Algorithm with Obstacle Constraints Based on Artificial Immune System

Computational intelligence techniques have been widely applied to data engineering research, including classification, clustering, deviation, or outlier detection [19]. Artificial immune system (AIS) is an intelligent method, which mimics natural biological function of the immune system. For its promising performance in immune recognition, the ability of immune learning and immune memory, AIS gradually becomes an important branch of intelligent computing [26–29]. In order to solve the problems of the traditional cluster algorithm in sensitivity to the initial value and the tendency to fall into local optimum, while maintaining its advantages of fast convergence speed, a novel spatial clustering algorithm with obstacle constraints is proposed in this paper.

#### 2.3.1. The Clustering Problem

Given *V*, the goal of a clustering algorithm is to obtain a partition *I* = {*I*_1_, *I*_2_,…, *I*_*k*_} (i.e., *I*_*i*_ ≠ *ϕ*, for all *i*; ⋃_*i*=1_^*k*^ *I*_*i*_ = *V*; *I*_*i*_∩*I*_*j*_ = *ϕ*, for all *i* ≠ *j*) which satisfies that objects classified as the same cluster are as similar to each other as possible, whereas objects classified as the different clusters are as dissimilar as possible.

#### 2.3.2. Antibody Encoding

Let *V* = {*v*_1_, *v*_2_,…, *v*_*M*_} be a set of *M* sample points, corresponding to the antigen set *Ags* = {*ag*_1_, *ag*_2_,…, *ag*_*M*_}. The antibody set *Abs* = {*ab*_1_, *ab*_2_,…, *ab*_*N*_}, where *N* is the number of antibodies. Each antibody *ab*_*i*_ consists of *k* cluster centers, and each cluster center can be expressed as a real-value *d*-dimensional profile vector which is represented as $ab_{i}=\{\underset{c_{1}}{\underset{︸}{a_{11}a_{12}\ldots a_{1d}}}\cdots \underset{c_{i}}{\underset{︸}{a_{i1}a_{i2}\ldots a_{id}}}\cdots \underset{c_{k}}{\underset{︸}{a_{k1}a_{k2}\ldots a_{kd}}}\}$, where *c*_*i*_ corresponds to the center of the *i*th-cluster.

#### 2.3.3. Affinity Function Design and Immune Operators

In most occasions, the most used similarity metric in a clustering algorithm is distance metric. The total within-cluster variance or the total mean-square quantization error (MSE) [30] is calculated as follows:

(1) $$\begin{array}{c} \text{Perf}\left(V,C\right)=\overset{m}{\underset{i=1}{\sum }}\left\{{\left\|v_{i}-c_{j}\right\|}^{2},\;\;j=1,\ldots ,k\right\}, \end{array}$$

where ‖*v*_*i*_ − *c*_*j*_‖ denotes the similarity between sample point *v*_*i*_ and clustering center *c*_*j*_ and the obstacle distance is used as a distance metric in this paper. Obstacles constraints should be taken into account for clustering algorithms in the paper. On this basis, cluster centers set *C* = {*c*_1_, *c*_2_,…, *c*_*k*_} and the corresponding partition *I* = {*I*_1_, *I*_2_,…, *I*_*k*_} are achieved by applying the rule that the nearer sample points are apart from a cluster center in obstacle distance.

Bearing in mind the measurement of the MSE in (1), we design an affinity function *f*_*i*,*j*_ in (2), which represents the affinity of the antibody of *i* with antigen *j*. Let *D*_in-cluster_ = ∑_*j*=1_^*k*^∑_*v*_*i*_∈*V*∩*I*_*j*__ *d*_*o*_(*v*_*i*_, *c*_*j*_); then

(2) $$\begin{array}{c} f_{i,j}=\frac{1}{D_{\text{in-cluster}}+\varepsilon_{0}}, \end{array}$$

where *ε*_0_ is a small positive number to avoid illness (i.e., denominator equals zero). *fmeans* denotes the average value of population affinity, which can be calculated as

(3) $$\begin{array}{c} fmeans=\frac{\sum_{i=1}^{k}\sum_{j=1}^{m}f_{i,j}}{k}. \end{array}$$

*M*⊆*Abs* is memory cell subset. Threshold value of immunosuppression is calculated as

(4) $$\begin{array}{c} \alpha =\frac{1}{k^{2}}\overset{k-1}{\underset{i=1}{\sum }}\overset{k}{\underset{j=i+1}{\sum }}f_{i,j}^{\prime }, \end{array}$$

where *f*_*i*,*j*_′ = *d*_*o*_(*c*_*i*_, *c*_*j*_), which represents the affinity of the antibody of *i* with antibody *j*.

The antibody selection operations, cloning operations, and mutation operations of AICOE algorithm were defined in the literature [31].

#### 2.3.4. Artificial Immune Clustering with Obstacle Entity (AICOE) Algorithm

For the antigen set *Ags* = {*ag*_1_, *ag*_2_,…, *ag*_*M*_}, the algorithm is described as follows.

*Step  1*. Initialize antibody set *Abs*(0) = {*ab*_1_, *ab*_2_,…, *ab*_*N*_}, where *N* is the number of antibodies. Consider *t* = 0.

*Step  2*. For all *ag*_*i*_ ∈ *I*_*k*_(1 ≤ *i* ≤ *M*, 1 ≤ *k* ≤ *N*), calculate the value of *f*_*i*,*k*_ according to (2).

*Step  3*. According to the affinity calculations by Step 2, optimal antibody subset *bstAS* is composed of top *K*(*K* ≤ *N*) affinity antibodies where *bstAS*⊆*Abs*(*t*). Add *bstAS* to *M*.

*Step  4*. Generation of the next generation antibody set is elaborated as follows.

- Obtain *bstAS*1 via performing clone operation on *bstAS*.
- Obtain *bstAS*2 via performing mutation operation on *bstAS*1. Add *bstAS*2 to *M*.
- Implement the immunosuppression operation on *M*. Calculate the value of *α* according to (4). For all *ab*_*i*_, *ab*_*i*_ ∈ *M*, if the value of *f*_*i*,*j*_′ is less than *α*, randomly delete one of the two antibodies.
- Randomly generate antibody subset to update the next generation antibody set, denoted by* rdmAS*.
- Add *M* and *rdmAS* to *Abs*(*t* + 1). Consider *t* = *t* + 1.

*Step  5*. Calculate the value of the *fmeans* of contemporary population by using (3). If the difference *fmeans* in certain continual iterations does not exceed *ε*, stop the algorithm; otherwise go to Step  2.

## 3. Case Implementation and Results

This paper presents two sets of experiments to prove the effectiveness of the AICOE algorithm. The first experiment uses a set of simulated data, which are generated by the simulation of ArcGIS 9.3. Experimental results are compared with *K*-means clustering algorithm [2, 3]. The second experiment is carried out on a case study on Wuhu city and compares the results with the COE-CLARANS algorithm [8]. All algorithms are implemented in C# language and executed on* Pentium* 4.3 HZ, 2 GB RAM computers. The main parameters of the algorithm are defined as follows: mutation rate *p*_*m*_ = 0.35, inhibition threshold *α* = 0.05, and the iterative stopping criteria parameter *ε* = 1.0*e* − 4.

### 3.1. Simulation Experimental Results

**Figure 3:**
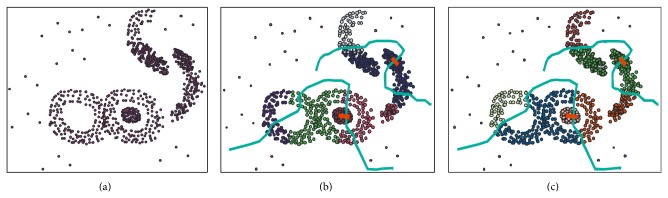
Clustering spatial points in the presence of obstacles and facilitators: (a) simulated dataset; (b) clustering results of *K*-means algorithm with obstacles and facilitators; (c) clustering results of AICOE algorithm with obstacles and facilitators.

The classical *K-*means clustering algorithm has been widely used for its simplicity and feasibility. The AICOE algorithm uses obstacle distance defined in this paper for clustering analysis, and *K-*means algorithm uses Euclidean distance as similarity measure of samples. Simulated dataset of the first experiment is shown in Figure 3(a). When cluster number *k* = 6, the clustering results of *K-*means clustering algorithm and AICOE algorithm are shown in Figures 3(b) and 3(c), respectively. Experimental results show that the clustering results of the AICOE algorithm considering obstacles and facilitators are more efficient than *K-*means algorithm.

### 3.2. A Case Study on Wuhu City

#### 3.2.1. Study Area and Data

**Figure 4:**
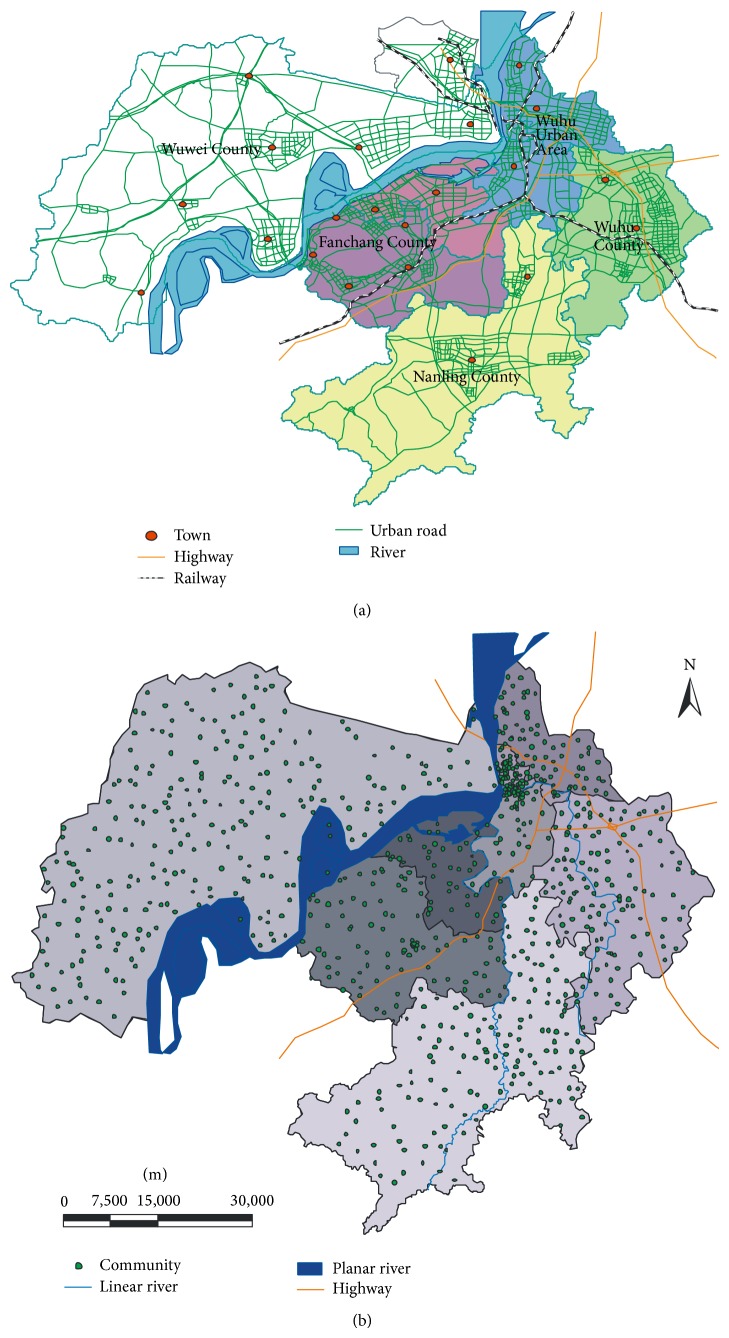
The spatial distribution of Wuhu city: (a) administrative map of Wuhu city; (b) the spatial distribution of communities in Wuhu.

In this test, the AICOE algorithm is applied to an urban spatial dataset of the city of Wuhu in China (Figure 4). This paper takes 994 residential communities as two-dimensional points, where the points are represented as (*x*, *y*). In this case study, each residential community is treated as cluster sample point, with its population being an attribute. The highways, rivers, and lakes in the territory are regarded as spatial obstacles, as defined in Definitions 1 and 2, respectively. Pedestrian bridge and underpass on a highway and the bridge on the water body serve as connected points, and the remaining vertices are unconnected points. Digital map of Chinese Wuhu stored in ArcGis 9.3 was used. And automatic programming has been devised to generate spatial points as cluster points to the address of the residential communities. The purpose of this paper is to find the suitable centers (medoids) and their corresponding clusters.

#### 3.2.2. Clustering Algorithm Application and Contrastive Analysis

**Figure 5:**
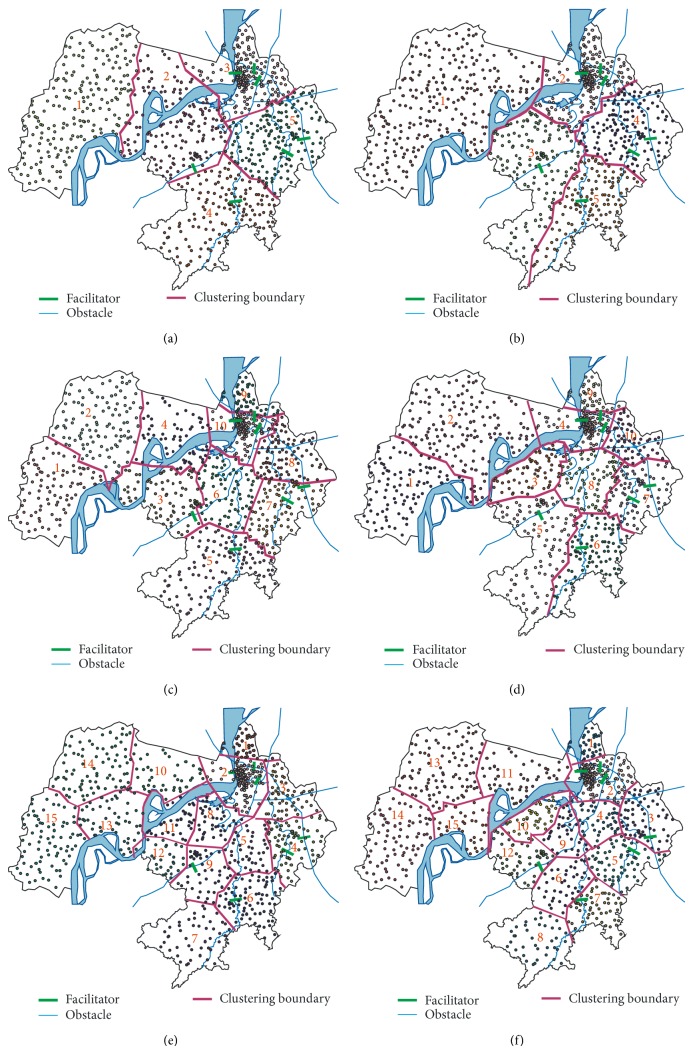
Comparison of clustering analysis using the COE-CLARANS algorithm and the AICOE algorithm: (a) 5 subclasses (COE-CLARANS algorithm); (b) 15 subclasses (AICOE algorithm); (c) 10 subclasses (COE-CLARANS algorithm); (d) 15 subclasses (AICOE algorithm); (e) 15 subclasses (COE-CLARANS algorithm); (f) 15 subclasses (AICOE algorithm).

**Figure 6:**
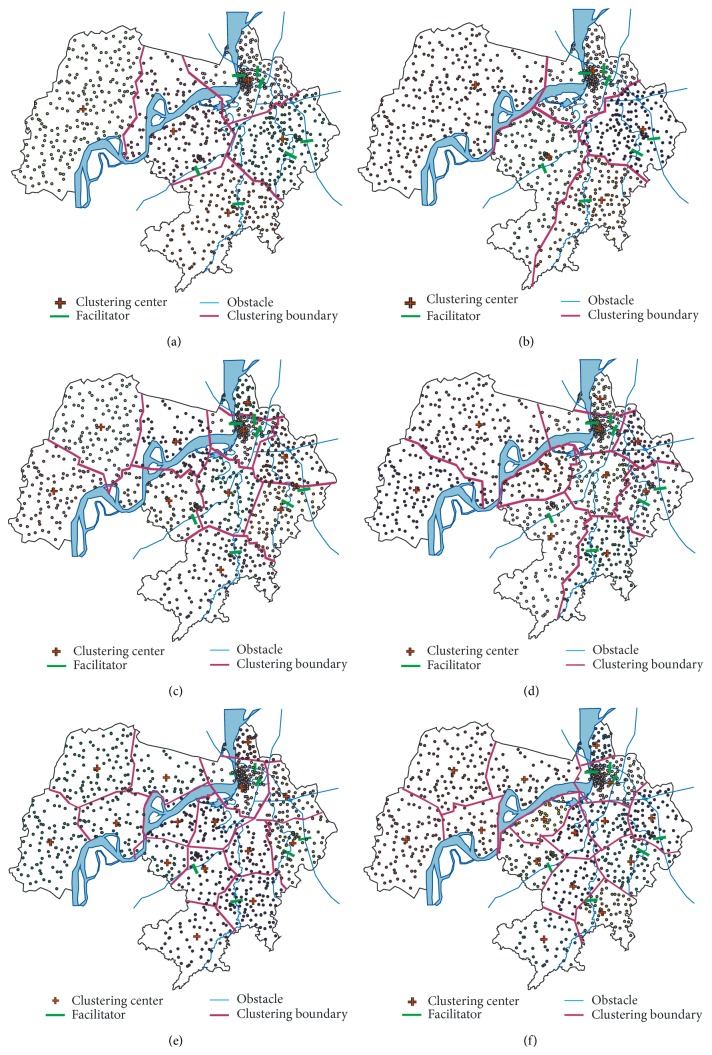
Comparison of clustering analysis using the COE-CLARANS algorithm and the AICOE algorithm considering clustering center: (a) 5 subclasses (COE-CLARANS algorithm); (b) 15 subclasses (AICOE algorithm); (c) 10 subclasses (COE-CLARANS algorithm); (d) 15 subclasses (AICOE algorithm); (e) 15 subclasses (COE-CLARANS algorithm); (f) 15 subclasses (AICOE algorithm).

The COE-CLARANS algorithm [8] and the AICOE algorithm are compared by simulation experiment. The AICOE algorithm uses obstacle distance defined in this paper for clustering analysis. The comparison results of clustering analysis using COE-CLARANS algorithm and AICOE algorithm are shown in Figure 5, and the comparison results of clustering analysis using COE-CLARANS algorithm and AICOE algorithm considering clustering centers are shown in Figure 6.

Given the covered range of different types of public facilities, a clustering simulation is carried out to generate 5, 10, and 15 subclasses, respectively, in this paper. Because Yangtze River is the main obstacle of Wuhu territory, the clustering result of its surrounding regions can demonstrate the validity of the algorithm. Setting cluster number *k* = 5, the clustering results of the AICOE algorithm show that only one clustered region 2 has been passed through by Yangtze River where Wuhu Yangtze River Bridge plays a role as a facilitator. While the clustering results of the COE-CLARANS algorithm show that Yangtze River has passed through two clusters, the clustered region 2 does not have any facilitators. Setting cluster number *k* = 10, the clustering results of the COE-CLARANS algorithm show that Yangtze River has passed through three subclass regions and the clustered regions 3 and 4 do not have any facilitators. Setting cluster number *k* = 15, there does not exist any facilitator in the clustered region 11 obtained by the COE-CLARANS algorithm. In comparison, the clustering results of the AICOE algorithm show that only one clustering region has been passed through by Yangtze River where the facilitator exists. The simulation results demonstrate that the impacts of obstacles on clustering results correspondingly reduce along with the increase in the number of cluster regions.

**Figure 7:**
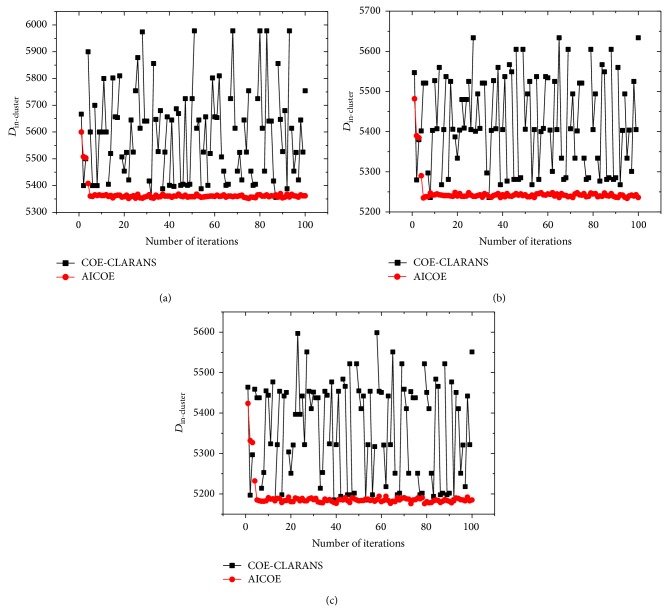
Comparison of clustering analysis using the COE-CLARANS algorithm and the AICOE algorithm by intercluster distances: (a) cluster number *k* = 5; (b) cluster number *k* = 10; (c) cluster number *k* = 15.

Figure 7 demonstrates that the COE-CLARANS algorithm is sensitive to initial value, while the AICOE algorithm avoids this flaw effectively. Meanwhile, the AICOE algorithm can get global optimal solution in fewer iterations.

Table 1 shows the results of scalability experiments for the comparison of the COE-CLARANS algorithm and the AICOE algorithm. The synthetic dataset in the following experiments is generated from a Gaussian distribution. The size of dataset varies from 25,000 to 100,000 points. The obstacles and facilitators are generated manually. The number of the obstacles varies from 5 to 20, and the number of vertices of each obstacle is 10. The number of the facilitators accounts for 20% of the number of the obstacles. Table 1 illustrates that the AICOE algorithm is faster than the COE-CLARANS algorithm.

By comparison of the COE-CLARANS algorithm and the AICOE algorithm for handling spatial clustering with physical constraints, the experimental results show that the COE-CLARANS algorithm causes grouping biases due to its microclustering approach. Correspondingly, the AICOE algorithm operates with all the data with less prior preprocessing. The quality of clustering results achieved by the AICOE algorithm surpasses the results of the COE-CLARANS algorithm. Next, the simulation results also indicate that the AICOE algorithm overcomes the COE-CLARANS shortcoming of sensitivity to initial value. The reason for this drawback is that COE-CLARANS algorithm selects the optimum set of representatives for clusters with a two-phase heuristic method. Last, the results of scalability experiments illuminate that the COE-CLARANS algorithm which is affected by the low efficiency of preprocessing runs slower than the AICOE algorithm.

## 4. Conclusions

Artificial immune clustering with obstacle entity algorithm (i.e., AICOE) has been presented in this paper. By means of experiments on both synthetic and real world datasets, the AICOE algorithm has the following advantages. First, through the path searching algorithm, obstacles and facilitators can be effectively considered with less prior preprocessing compared to the related algorithm (e.g., COE-CLARANS). Then, by embedding the obstacle distance metric into affinity function calculation of immune clonal optimization and updating the cluster centers based on the elite antibodies, the AICOE algorithm effectively solves the shortcomings of the traditional method. The comparative experimental and case study with the classic clustering algorithms has demonstrated the rationality, performance, and practical applicability of the AICOE algorithm.

Due to the complexity of geographic data and the difference of data formats, present researches on spatial clustering with obstacle constraint mainly aim at clustering method for two-dimensional spatial data points [8, 10, 12–14]. There are two directions for future work. One is to extend our approach for conducting comprehensive experiments on more complex databases from real application. The other is to take nonspatial attributes into account for a comprehensive analysis of spatial database.

## Figures and Tables

**Table 1:** Run time comparison of COE-CLARANS and AICOE (seconds).

Number of points	5 obstacles (50 vertices)		10 obstacles (100 vertices)		20 obstacles (200 vertices)	
	COE-CLARANS	AICOE	COE-CLARANS	AICOE	COE-CLARANS	AICOE
25 k	25.86	20.58	31.54	25.69	36.92	26.87
50 k	43.16	31.59	46.85	35.67	49.36	36.98
75 k	58.22	39.56	61.23	41.23	63.33	42.36
100 k	82.63	64.55	83.79	65.32	83.94	65.87
